# Supplementary material for: Long-Range Ionic and Short-Range Hydration Effects Govern Strongly Anisotropic Clay Nanoparticle Interactions
Source: J Phys Chem C Nanomater Interfaces. 2022 May 3;126(18):8143–51. doi: 10.1021/acs.jpcc.2c01306 (PMC9109138; doi:10.1021/acs.jpcc.2c01306)
Supplement: Supplementary file 1 — jp2c01306_si_001.pdf [file jp2c01306_si_001.pdf]

Supporting Information for: Long Range Ionic And Short Range Hydration Effects Govern Strongly Anisotropic Clay Nanoparticle Interactions

Andrea Zen,[1,2,3,*] Tai Bui,[3,4,5,*] Tran Thi Bao Le,[6] Weparn J. Tay,[5] Kuhan Chellappah,[5] Ian R. Collins,[5] Richard D. Rickman,[5] Alberto Striolo,[6,7] and Angelos Michaelides[8,3,4,†]

[1]*Dipartimento di Fisica Ettore Pancini, Università di Napoli Federico II, Monte S. Angelo, I-80126 Napoli, Italy*

[2]*Department of Earth Sciences, University College London, Gower Street, London WC1E 6BT, United Kingdom*

[3]*Thomas Young Centre and London Centre for Nanotechnology, 17–19 Gordon Street, London, WC1H 0AH, United Kingdom*[‡]

[4]*Department of Physics and Astronomy, University College London, Gower Street, London WC1E 6BT, United Kingdom*

[5]*BP Exploration Operating Co. Ltd, Chertsey Road, Sunbury-on-Thames TW16 7LN, United Kingdom*

[6]*Department of Chemical Engineering, University College London, WC1E 7JE, London, United Kingdom*

[7]*School of Chemical, Biological and Materials Engineering, University of Oklahoma, Norman, OK 73019, United States*

[8]*Yusuf Hamied Department of Chemistry, University of Cambridge, Lensfield Road, Cambridge CB2 1EW, United Kingdom*

(Dated: April 13, 2022)

* These two authors contributed equally
† am452@cam.ac.uk
‡ andrea.zen@unina.it

CONTENTS

S1.	The structure of simulated kaolinite nanoparticles	S4
S2.	Density functional theory tests	S6
S3.	Umbrella sampling simulation protocol	S9
S4.	Temperature dependence of PMF	S11
S5.	PMF per-surface-area profiles	S12
S6.	Identification of free energy wells and barriers	S13
S7.	Particle-particle interactions	S15
S8.	Solvation free energy	S16
S9.	Density profiles of water oxygen and hydrogen atoms	S18
S10.	Density of water	S20
S11.	Hydrogen bonds	S21
S12.	Diffusion coefficient of water	S22
S13.	Particle dynamics in solution	S23
	References	S23

S1. THE STRUCTURE OF SIMULATED KAOLINITE NANOPARTICLES

The bulk structure of kaolinite is known and well characterised. For the present work, we took as reference the crystallographic structure of Bish [1] [2]. The relaxed structures obtained using the chosen ClayFF force field or using DFT-based approaches are in close agreement with the crystallographic experimental bulk structure of kaolinite. The crystallographic structure of kaolinite nanoparticles is instead not immediately available. However, from the literature there are two very stable basal surfaces, and the edge surfaces correspond to the following planes: (0 1 0), (0 -1 0), (1 1 0), (-1 -1 0), (1 -1 0), and (-1 1 0). Cleavage along these planes has also been studied in the literature, using force fields and DFT, and precise indications on the cleaving and hydrogenation of dangling bonds are provided, for instance, in [3] and references therein, resulting in five-fold coordination of edge Al atoms. Here we followed those prescriptions, and considered a 3-layer ($\sim$ 20 Å) hexagonal kaolinite nanoparticle with side lengths of $\sim$ 18 Å. The nanoparticle was created in the the VESTA package [4] and dangling bonds on edges were hydrogenated using a Python script written explicitly to fulfil the indications given in the literature. The surface area for each edge is roughly $A_{\text{edge}} \sim 18 \times 20 = 360$ Å^2, and the surface area of the basal surfaces is roughly $A_{\text{face}} \sim \frac{3\sqrt{3}}{2} \times 18^2 \sim 842$ Å^2. The ratio between the face and the edge surface of the simulated particles is $\frac{A_{\text{edge}}}{A_{\text{edge}}} \sim 2.3$. We expect that face-to-face interfaces are, in a first approximation, proportional to A_{face} and the face-to-edge interfaces are proportional to A_{edge}.

The results shown in the manuscript are obtained from classical molecular dynamics simulations employing semi-empirical force fields, are discussed in the computational details. In each simulation cell there are two kaolinite nanoparticles and 10,000 to 11,000 water molecules. In line with ref. [5], the simulation cell is roughly $60 \times 60 \times 100$ Å^3 for PMF calculations of face-to-face orientation, and $60 \times 60 \times 110$ Å^3 for PMFs calculations of face-to-edge orientation. Periodic boundary conditions are employed in all three dimensions. An image of the nanoparticles in face to face configuration is shown in Figure S1.

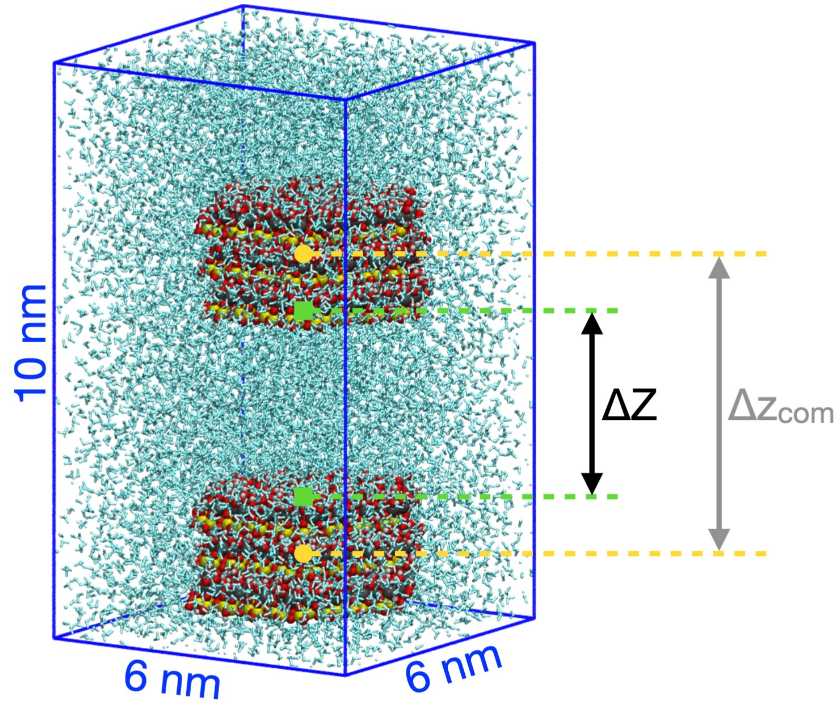

FIG. S1. A representative simulation snapshot of the face-to-face case in the gibbsite-siloxane orientation. The blue box is the simulation cell, where periodic boundary conditions are employed. Inside the box, two nanoparticles are surrounded by 10,000 water molecules, coloured in cyan. Other colors are the same as those shown in Figure 1 in the manuscript. The distance among the two nanoparticles is evaluated during the MD simulation by measuring the projection on the z-axis of the distance among the center of mass of the heavy atoms in the two nanoparticles, which is indicated as Δz_{com} in the image. The umbrella sampling constraints in our simulations are enforced on Δz_{com}, and in this specific orientation we performed MD simulations with Δz_{com} ranging from 2 nm to 5 nm. The PMF curves have been plotted as functions of ΔZ, which is the minimum distance among the heavy atoms in the two particles, because it facilitates the comparison among the curves of different orientations. Indeed, Δz_{com} and ΔZ differ by an offset, which depends on the specific orientation among the nanoparticles.

S2. DENSITY FUNCTIONAL THEORY TESTS

In order to validate the reliability of the force field parametrization employed in the present work (described in the methods section of the manuscript), we performed a set of simulations using *ab-initio* approaches. Among the latter methods, the most affordable is density functional theory (DFT). DFT is able to provide evaluations in good agreement with experiment or higher level of theories, as quamtum Monte Carlo, in systems including clay, water and ions, as long as it employs a correction for the Van der Waals (vdW) [6]. There are many different flavours of DFT and of associated vdW-correction schemes, and some setups proves more reliable than others. Based on some benchmarks reported in [6], we selected a good setup for DFT and we performed DFT-based geometry relaxations and MD simulations of the kaolinite edges and faces, to simulate the adsorption of water, sodium or chlorine ions. For instance, minimum energy structures obtained from our DFT simulations of chlorine and sodium ions on the basal surfaces of kaolinite are shown in figure S2.

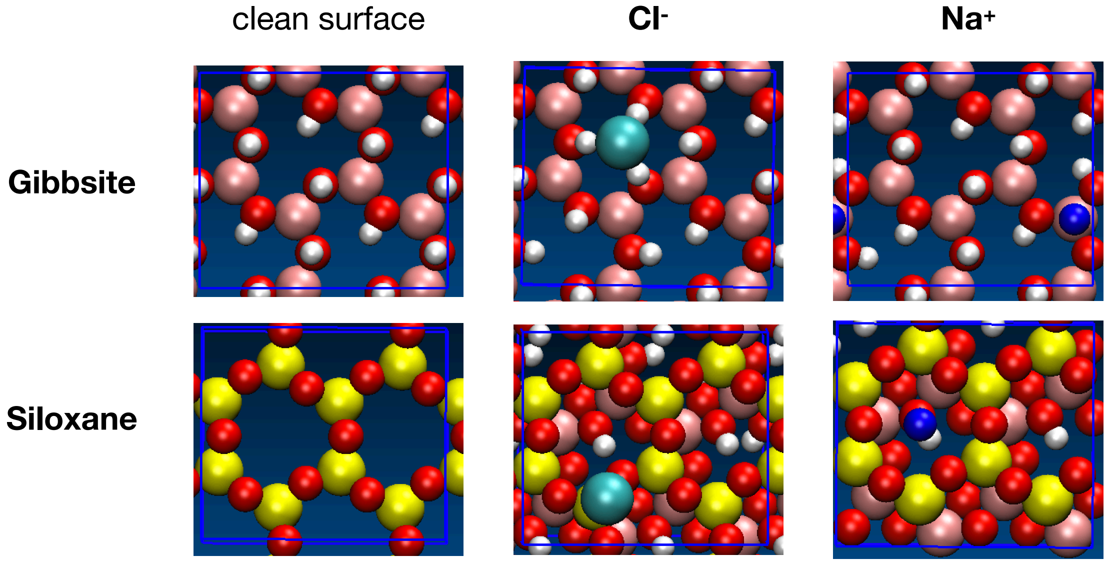

FIG. S2. The images show a top view of the minimum energy structures, as obtained from our DFT simulations, of the clean gibbsite surface (top left), a chlorine ion on the gibbsite surface (top conter), a sodium ion on the gibbsite surface (top right), the clean siloxane surface (bottom left), a chlorine ion on the siloxane surface (bottom conter), a sodium ion on the siloxane surface (bottom right). Hydrogen, oxygen, aluminium, silicon, chlorine and sodium atomic species are coloured in white, red, pink, yellow, cyan and blue, respectively.

Adsorption was examined on a single layer of kaolinite, a system with 2D periodicity along the A and B axes. The simulated supercell was 1 × 2 the conventional unit cell of bulk kaolinite (ca. 10.38 Å× 9.01 Å) and is comprised of 8 aluminium, 8 silicons, 36 oxygen, and 16 hydrogen

atoms for the kaolinite slab, plus the atoms of the adsorbed molecule or ion. We used a C axis of 22.25 Å, yielding ca. 15 Å of vacuum in the direction orthogonal to the slab. We relaxed the atomic configurations, keeping the cell axes fixed, in all the systems under consideration: the free standing slab, and the slab plus the adsorbate on either the gibbsite or the siloxane face, being the adsorbate either a water molecule, a Na^+ ion or a Cl^- ion. DFT simulations were performed with the CP2K [7] software package using the Quickstep algorithm, the PBE-D3(BJ) [8–10] functional, Goedecker-Teter-Hutter pseudopotentials [11, 12], a 1000 Ry plane-wave cut-off, a relative cut-off of 60 Ry, TZVP molecularly optimised basis set [13] on H, O, and Si, and DZVP molecularly optimised basis set on Al and Cl (because the TZVP was not available). The plane-wave cutoffs were carefully tested on the adsorption of water on both faces of kaolinite, where the basis set error with the selected basis set is < 1 meV. It was not possible to make a comparable convergence test on the localised basis sets, as some atomic species only the SZV and the DZVP are provided. Thus, we compared the adsorption energy of water on both surfaces with the results from the VASP [14] package, using the same force field, the PAW potentials [15] and converged plane wave cutoffs. The VASP and CP2K results agree within 1 kcal/mol. In the simulations where the adsorbate is an ion the overall system is charged, either positively or negatively, so a uniform compensating background charge is added, as it is standard practice in DFT.

Each adsorption energy $E_{\rm ads}$ was estimated as the difference between the energy of the optimised configuration $E_{\rm opt}$ and the energy of the systems far away $E_{\rm far}$: $E_{\rm ads} = E_{\rm opt} - E_{\rm far}$. In particular, $E_{\rm far}$ is the energy of the system of the relaxed structure of the free standing slab plus the adsorbate in the middle of the vacuum region (i.e., over 7 Å away from both the faces) [16]. The $E_{\rm ads}$ computed in this way are reported in Table S1. They are compared with the values obtained employing the classical force field setup that we employed in the rest of the project, namely ClayFF and the SPC/E water model. In the FF approach we used the same simulation cell as in DFT, but we relaxed the atomic positions. The DFT and the FF relaxed structures are in good agreement: they is a qualitatively good matching, and the main differences are little interatomic deviations.

The values of $E_{\rm ads}$ computed using ClayFF+SPC/E seem to always underestimate the interaction strength if compared with DFT, however it yields to right ranking. It is worth to consider how much the DFT results depend on the particular setup that we have used. In this respect, the values from VASP, using the same PBE-D3 functional, gives -17.7 kcal/mol and -5.7 kcal/mol on W@G and W@S, respectively. This shows that the cutoffs are at convergence and there is little dependence on the employed pseudopotentials. The uncertainty due to the choice of the functional and the vdW correction is much larger, as shown for instance in Zen *et al.* [6]. Ref. [6] also shows

TABLE S1. Adsorption energy E_{ads}, in kcal/mol, of a water (W) molecule, a chlorine (Cl) ion or a sodium (Na) ion on either the gibbsite (G) or the siloxane (S) faces. Values have been obtained using the LAMMPS package [17] on the classical force field ClayFF and the SPC/E water model, and using the CP2K package [7] on DFT simulations with the PBE-D3 [8–10] functional (further details on the setup are reported on the text).

System	Classical FF ClayFF+SPC/E	DFT PBE-D3
W@G	-10.7	-17.6
W@S	-2.8	-6.1
Cl@G	-51.1	-64.1
Cl@S	-5.0	-6.2
Na@G	-31.8	-40.8
Na@S	-51.4	-64.3

that more accurate (and much more expensive) ab-initio approaches, such as quantum Monte Carlo, indicate the available vdW-corrected functionals are indeed underestimating E_{ads} of water on kaolinite, despite being definitely more reliable that DFT approached with no vdW correction, which always underestimate the adsorption. As a comparison, E_{ads} from the PBE functional is -13.2 kcal/mol and -2.3 kcal/mol on W@G and W@S, respectively.

In view of the above considerations, it appears that the level of reliability of the atomistic force field modelling available through ClayFF is appropriate for the purposes of this manuscript.

S3. UMBRELLA SAMPLING SIMULATION PROTOCOL

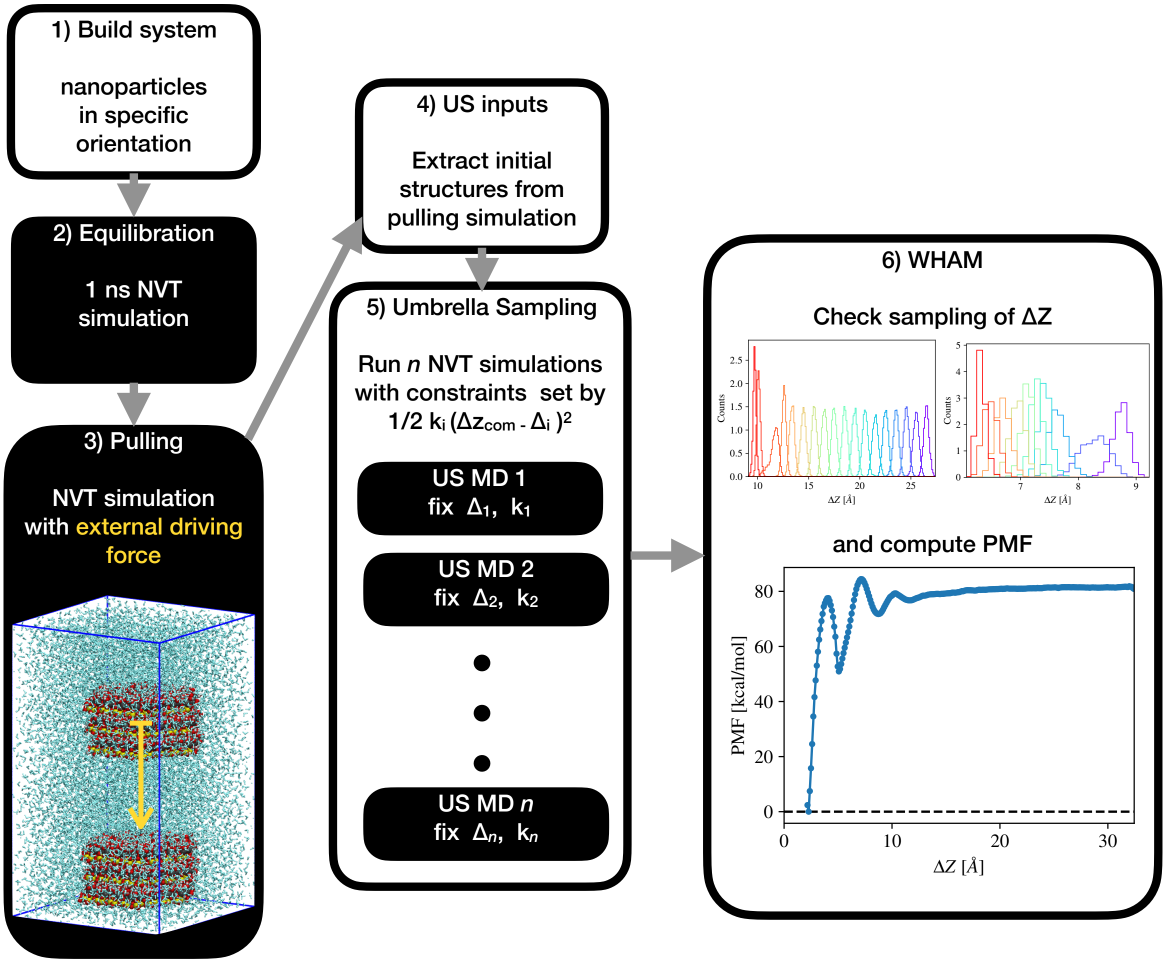

FIG. S3. Protocol that we developed and used to evaluate the PMF of two kaolinite nanoparticles. The first step is to decide the relative orientation of the nanoparticles, and generate the corresponding system. The second step is the equilibration of the system, in the NVT ensemble, for at least 1 ns. The third step is to introduce an external driving force on the upper nanoparticle driving it from far away to very close to the lower nanoparticle. We set this external force in order to move the nanoparticle 1 nm each ns. We are interested in a region of ΔZ ranging from 0 to 3 nm, thus step 3 requires 3 ns of MD simulation. In step 4, a subset of the configurations sampled in the pulling dynamics are chosen and used as starting configurations of n umbrella sampling (US) simulations. Each US simulation samples the phase space on a specific predefined interparticle distance. This is achieved by imposing to Δz_{com} a harmonic force potential with force constant k_i and center Δ_i. In step 5 the n US MD simulations are performed in an NVT ensemble. Finally, the outputs of the n US simulations are used to check the sampling of the ΔZ and build the PMF, using the Weighted Histogram Analysis Method (WHAM). Black boxes in the image correspond to running MD simulations. Further details are given in section S3.

The first step of the scheme was performed using the Packmol package [18] to create the structure of the two nanoparticles in a specific orientation and distance, and of the water molecules around them. Afterwards, we used the Moltemplate package [19] to generate the LAMMPS inputs. The

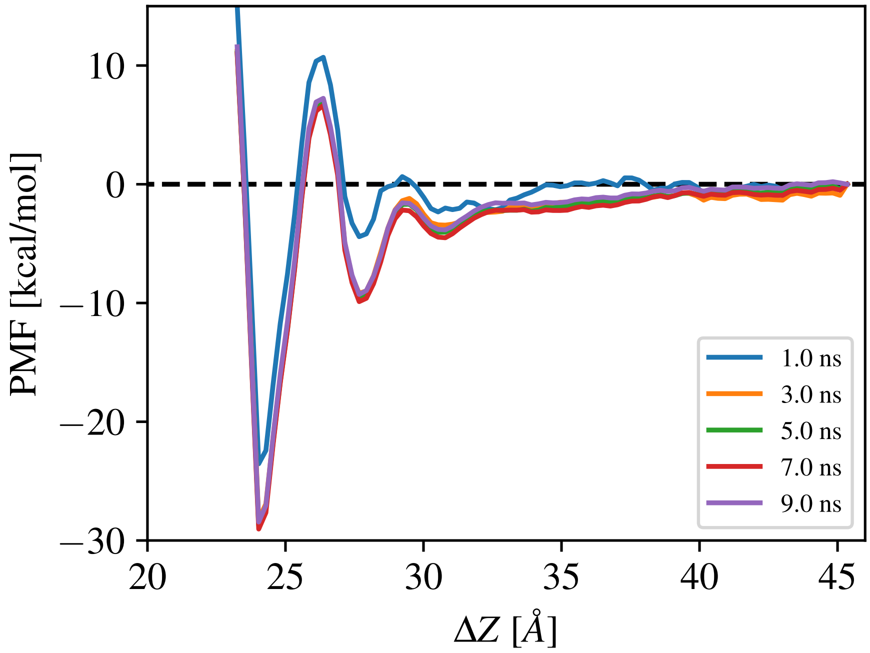

FIG. S4. The plot shows the predicted PMF curves obtained using increasingly long US MD simulations. The legend indicates the length of each US simulation used to obtain the corresponding curve. It can be seen that by ca. 3-5 ns the PMF profiles are converges with respect to simulation length.

MD simulations described in steps 2 and 3 and 5 have been performed in several computer clusters. We have checked that they can be performed efficiently using approx. 100 CPU cores. The pulling MD simulation in step 3 is aimed at providing good initial configurations for the following US simulations. The pulling has to be slow enough that the water particles have time to equilibrate around the nanoparticles. A drift velocity of 1 nm/ns was observed to be good. In step 4 a decision on how many US simulations to perform has to be made, which is related to the choice of force constant values k_i and the separation (i.e., the value of $|\Delta_i - \Delta_{i+1}|$) among them. We tested that an effective choice is to use a different setup in the regions corresponding to ΔZ larger and smaller of ca. 1 nm. Indeed, the PMF is quite smooth when the nanoparticles are separated by more than 1 nm, and we used a k_i of 10 kcal/mol and a separation $|\Delta_i - \Delta_{i+1}|$ of around 0.7 Å. When the nanoparticles are close the PMF can have steep oscillations, so we used a k_i of 50 kcal/mol and a separation $|\Delta_i - \Delta_{i+1}|$ of 0.2 Å.

S4. TEMPERATURE DEPENDENCE OF PMF

We consider here the dependence on the temperature of the PMF profiles. All the PMFs considered in the previous figures have been obtained at a temperature T=350 K. We performed an additional PMF evaluation at an higher temperature, namely the gibbsite-siloxane orientation at T=450 K. The comparison between the PMFs at T=350 K and T=450 K is shown in figure S5.

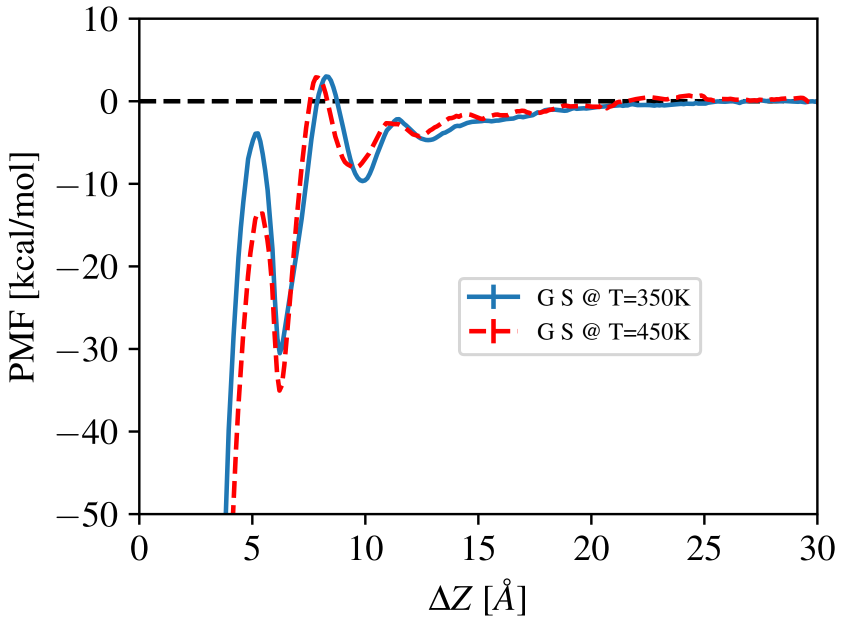

FIG. S5. PMF curves of the gibbsite-siloxane orientation at the temperature of T=350 K (blue) and T=450 K (red).

The comparison indicates that there is not a big sensitivity of the PMF on the temperature, at least for this specific orientation and specific modelling approach employed. The local minima and the barriers are only slightly different at the two temperatures considered.

S5. PMF PER-SURFACE-AREA PROFILES

Since kaolinite nanoparticles can have different sizes from those simulated here, in Figure S6 we have plotted the PMF per surface area. We cannot assume a precise linear dependence between the PMF and the surface area at the interface, because the borders of the interface are not negligible compared to the interface itself. However, in the first instance the assumption of linearity could be considered acceptable. Assuming it, the comparison of the curves in Figure S6 indicates that the "flatness" of the face-to-edge interfaces does not follow only the interface surface area and is an intrinsic property of the nanoparticle interactions.

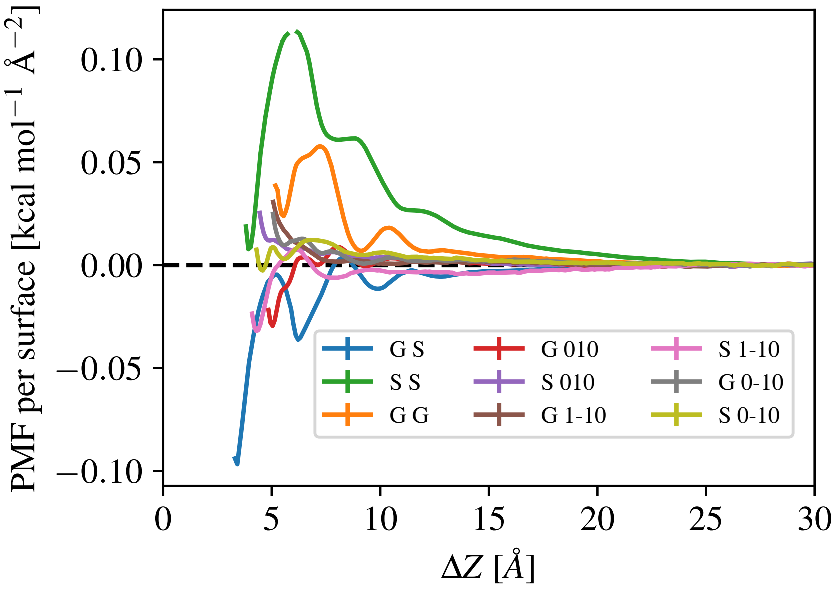

FIG. S6. PMF per surface area of all the orientations considered in this study, obtained from umbrella sampling simulations in the NVT ensemble at a temperature of 350 K, employing the ClayFF atomic force field for the kaolinite particles and the SPC/E water model. Different colours correspond to different orientations among the nanoparticles, as indicated in the legend, where G indicates the gibbsite basal surface, S the siloxane basal surface, and the numbers indicate the Miller indices of the corresponding edge. The abscissa of the figure reports the distance ΔZ, which is the minimum distance among the "heavy" atoms (i.e., Al or Si atomic species) of the two nanoparticles.

S6. IDENTIFICATION OF FREE ENERGY WELLS AND BARRIERS

Analysis of free energy profiles was conducted as shown in Figure S7. In this figure, the panels on the left illustrate the relationship between the actual and constrained distances between the two particles, which is used to identify the locations of the free energy wells and peaks. If a free energy minimum exists, the actual and constrained distances should be equal, while adjacent windows should have identical actual distances. Thus, the energy minima are identified as the points where the nearly horizontal portion of the ΔZ - ΔZ_0 plot intersects the diagonal line. On the other hand, the maxima's positions would be determined by a jump in the dZ values of the two adjacent windows. The positions of the free energy minima and maxima are depicted as vertical green and orange lines.

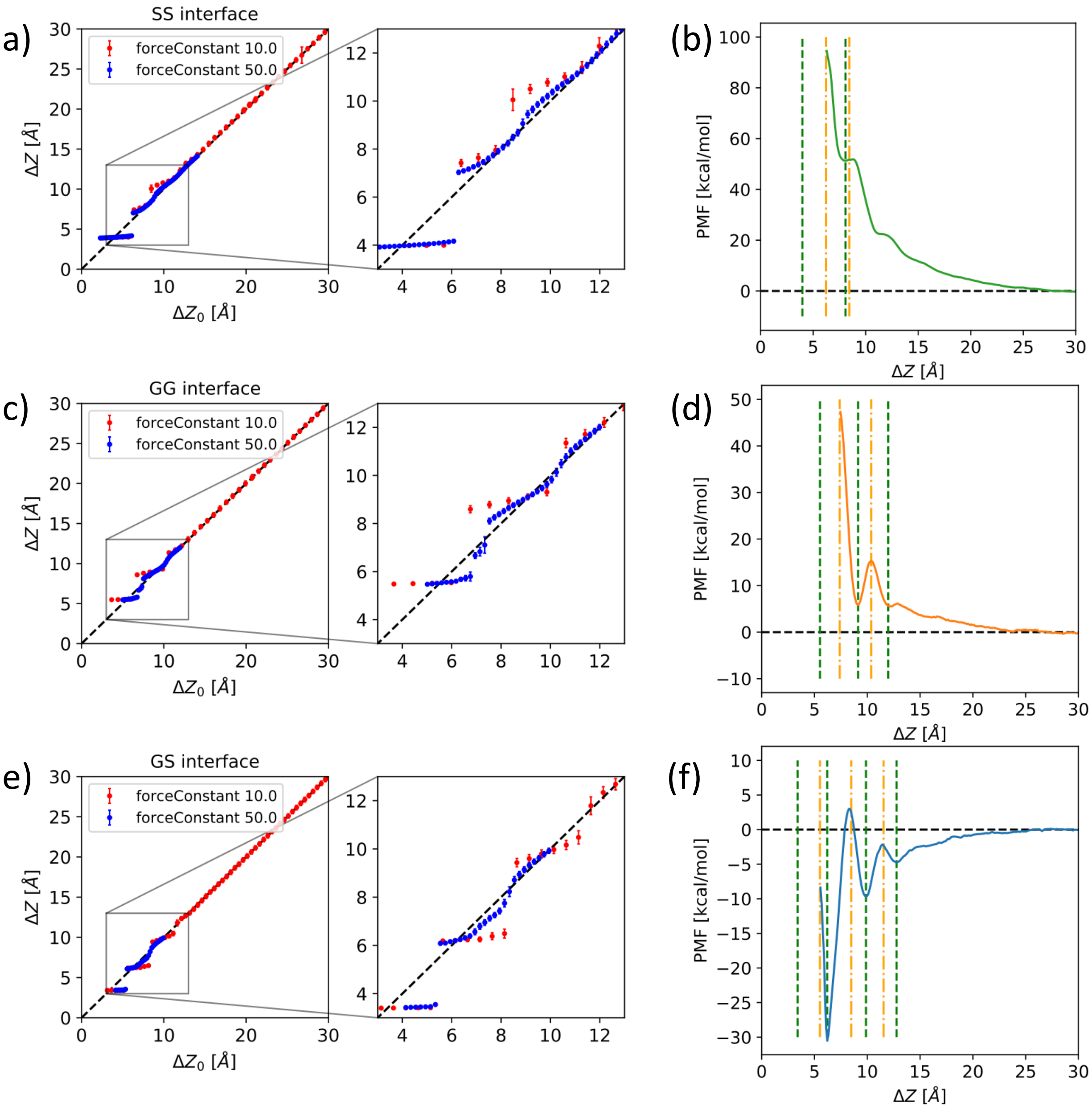

FIG. S7. (a, c, e) Relationship between the actual (ΔZ) and constrained (ΔZ_0) distances between two kaolinite particles from umbrella simulations for the siloxane-siloxane, gibbsite-gibbsite, and gibbsite-siloxane orientations, respectively. Inset plots are also shown on the right. (b, d, f) PMF profiles with locations of free energy wells (green) and barriers (orange) identified from assessing changes in the values of the (ΔZ, ΔZ_0) between two adjacent windows.

S7. PARTICLE-PARTICLE INTERACTIONS

In Figure S8 we plot the decomposition of particle-particale interaction into vdW and Coulomb contributions using `fix group/group` command implemented in LAMMPS[17]. Due to the similar charge distributions of the two approaching surfaces, the results show a strong repulsive Coulomb interaction between the two particles, which is opposite to what has been observed in the case of the gibbsite-siloxane orientation (see Figure 3 of the main text).

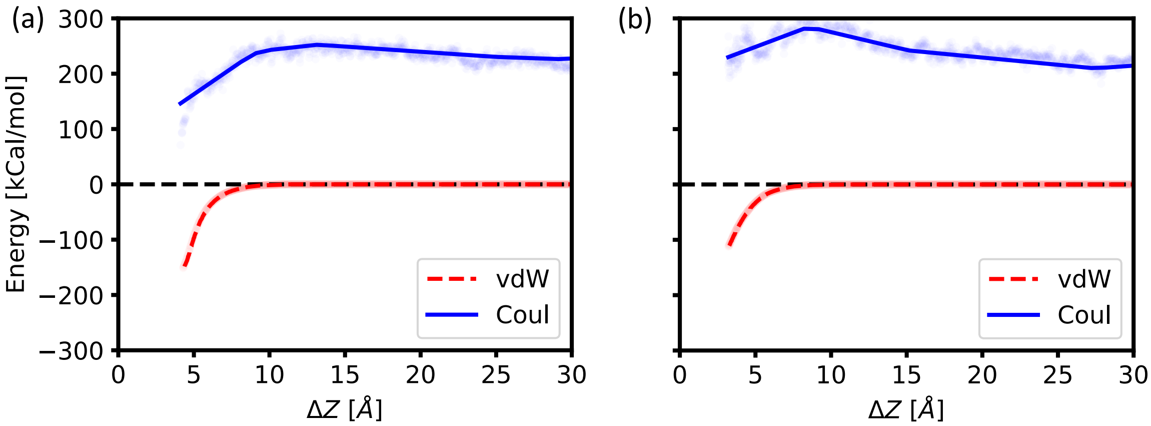

FIG. S8. Results of decomposition of particle-particle interactions: vdW and Coulomb potentials as a function of the distance between the two particles, calculated for siloxane-siloxane (a) and gibbsite-gibbsite (b) orientations, respectively. Noting that the Coulomb potential values are highly positive in both cases due to symmetrical charge distributions on the two approaching particles.

S8. SOLVATION FREE ENERGY

To obtain the solvation free energy, we applied the method described in [20–22], which is based on the quantification of time-averaged water density fluctuations. These are calculated via the probability of observing N molecules within a 6.6 Å diameter spherical volume, v, located at the center of the first hydration film.

$$P_v(N) = \langle \delta(N_v - N) \rangle = \lim_{t \to \infty} \left[\frac{1}{t} \int_0^t dt' \langle \delta(N_v(t') - N) \rangle \right] \tag{1}$$

Then the solvation free energy is calculated based on the relation:

$$\Delta\mu_v = -k_B T \ln P_v(0) \tag{2}$$

Figure S9 depicts the solvation free energies at various distances. Consistently with the other results presented in this manuscript, we observed a noticeable difference in the solvation free energy between different particle-particle separations in the cases of siloxane-siloxane and gibbsite-gibbsites orientations. Whereas, the solvation free energy obtained in the case of gibbsite-siloxane appears to remain constant (see Figure S9c, f, and g). The minimal change in the calculated solvation free energy indicates agglomeration along the gibbsite-siloxane orientation incurs a lower energy cost than along the gibbsite-gibbsite and siloxane-siloxane orientations.

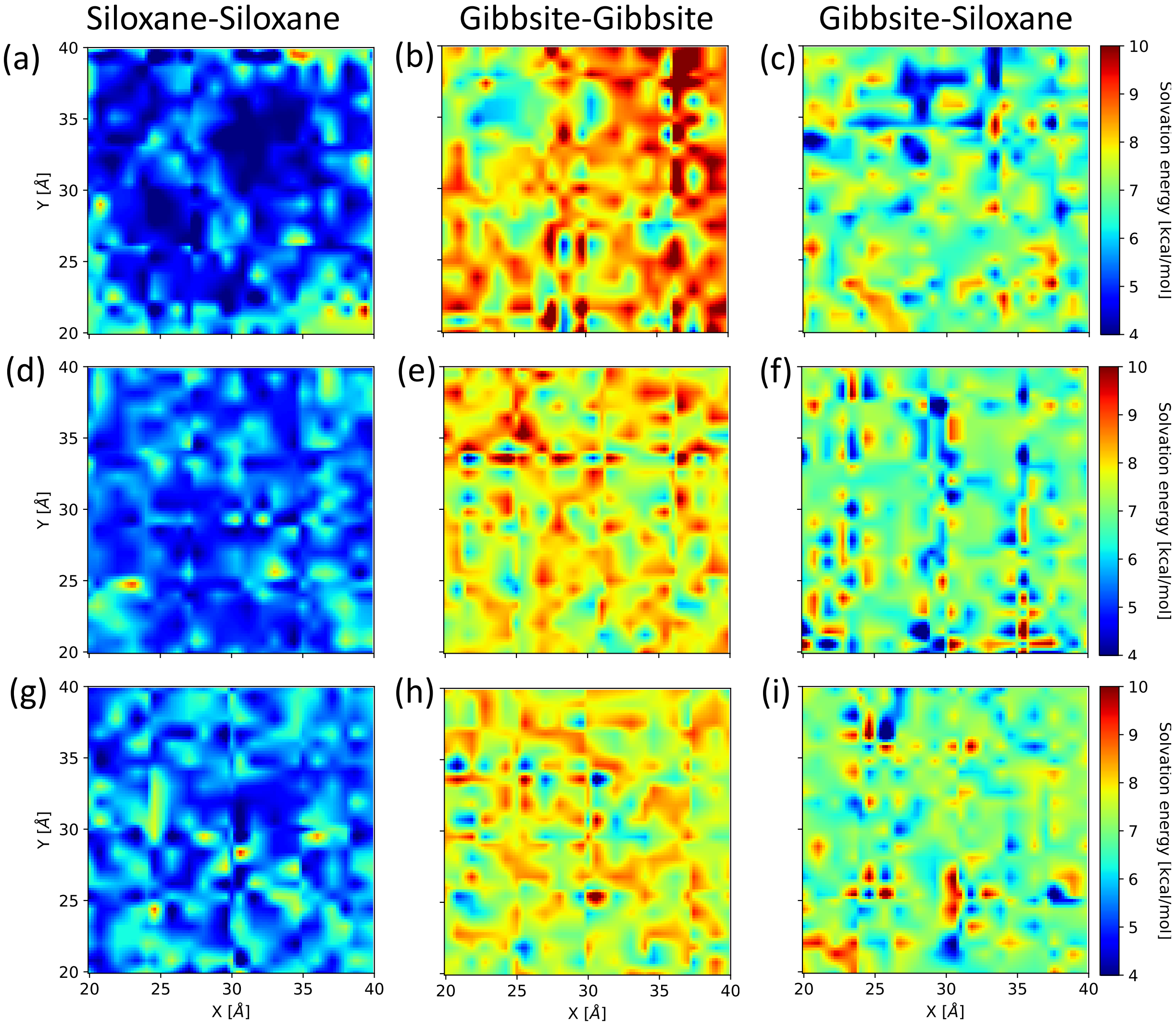

FIG. S9. In-plane solvation free energy distributions obtained for the siloxane and gibbsite surfaces in the siloxane-siloxane (SS), gibbsite-gibbsite (GG), and gibbsite-siloxane (GS) orientated systems. The results were obtained from density fluctuations of water within a 6.6 Å spherical volume located in middle of the first hydration layer of the siloxane and gibbsite surfaces (along the X-Y plane).

S9. DENSITY PROFILES OF WATER OXYGEN AND HYDROGEN ATOMS

Figure S10 shows the density of water oxygens and hydrogen atoms along the Z direction of the simulation box (perpendicular to the basal planes of the kaolinite particles) at various separation distances, as obtained from the MD simulations in pure water solution. The upper two profiles correspond to the local minima of the PMF.

Figure S10(a) for siloxane-siloxane shows that the density of the oxygen atoms is bimodal despite the interface being large enough to accommodate only one layer of water. The density profiles of hydrogen atoms in water molecules in Figure S10(a) exhibits three closely spaced peaks, with the middle peak roughly two times as large as the side peaks.

In contrast, in the case of gibbsite-gibbsite, we observed in Figure S10(b) that the density peaks of water oxygen atoms merge into a single sharp peak and the hydrogen atoms density is again three-modal. Based on the positions of the hydrogen and oxygen peaks, we infer that one of the water molecules' OH groups points toward the two surfaces and one is parallel to the surface. It is worth noting that the density profiles for siloxane-siloxane and gibbsite-gibbsite are highly symmetrical, as comes from the up-down inversion symmetry of the system.

In the case of gibbsite-siloxane orientation, Figure S10(c) shows asymmetric peaks; the oxygen atom density is unimodal but skewed, and the hydrogen atom density has one little peak close to the gibbsite surface and a big peak close to siloxane. These distributions are qualitatively similar to those of oxygen and hydrogen atoms in the proximity of gibbsite in the large ΔZ case (see Figure S10h and S10i). This results in a minimal cost required for merging the two hydration films at close inter-particle distances, which is consistent with the results calculated for water orientation (see Figure 4 in the manuscript).

These results indicate that in the cases of siloxane-siloxane and gibbsite-gibbsite orientations, there is a significant change in the relative distribution of water oxygen and hydrogen atoms within the hydration layers as the two particles approach each other. In contrast, we do not observe such a significant change in the case of gibbsite-siloxane orientation. This yields a negligible energy cost for merging the two hydration films at close inter-particle distances for the gibbsite-siloxane orientation, but a penalty for the siloxane-siloxane and gibbsite-gibbsite orientations. This is consistent with the analysis of the water orientation (see Figure 4 in the manuscript and the corresponding discussion).

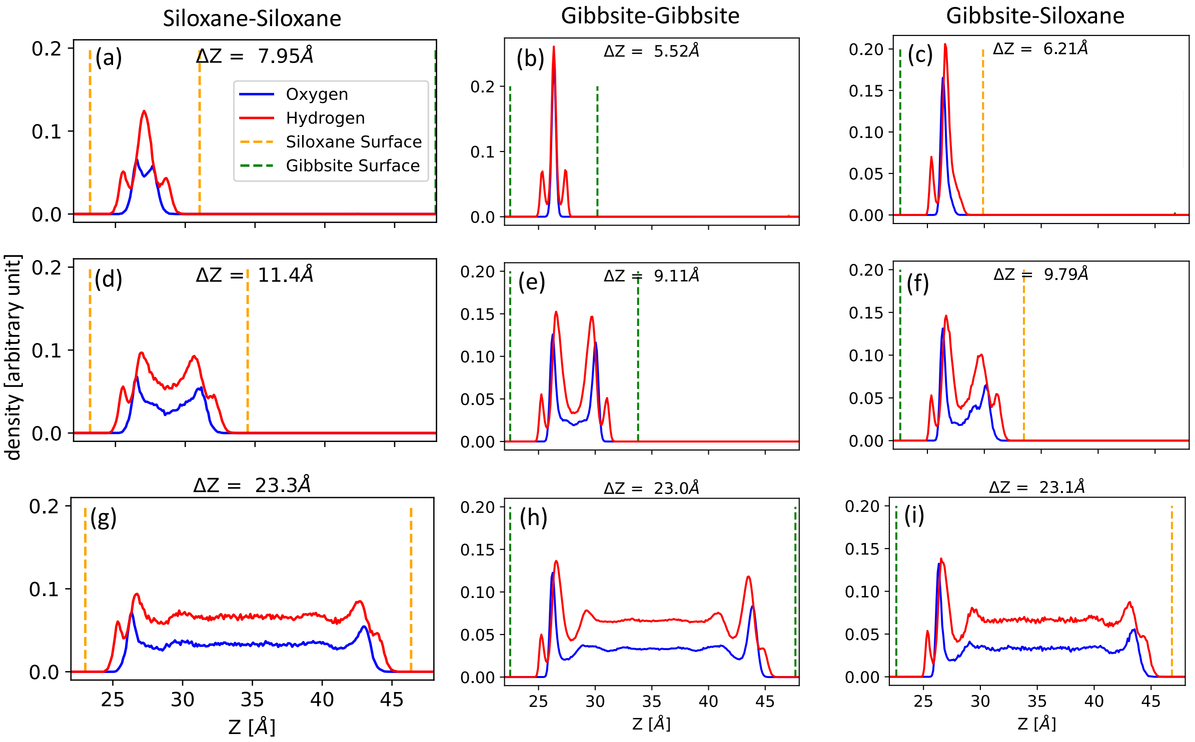

FIG. S10. Interface for simulations with pure water. (a)-(i) From left to right: density profile of water oxygen and hydrogen atoms along the Z direction of the box calculated for the siloxane-siloxane, gibbsite-gibbsite, and gibbsite-siloxane orientations, respectively. Density profiles obtained for positions of the energy wells are displayed from top to bottom.

S10. DENSITY OF WATER

We consider here the average density of water molecules between two approaching particles as a function of particle-particle separation. The results, shown in Figure S11, indicate that when the approaching surfaces contain at least one gibbsite surface (i.e., gibbsite-siloxane and gibbsite-gibbsite), the positions of the free energy wells correspond to the distances at which the number of confined water molecules increases. This is almost certainly due to the fact that water molecules prefer to form hydrogen bonds with gibbsite surfaces (see Figure S12). In the siloxane-siloxane orientation, on the other hand, water-enriched regions correspond to energy barriers. This could be because the siloxane surfaces are more hydrophobic, which do not support increased water density within the hydration layer.

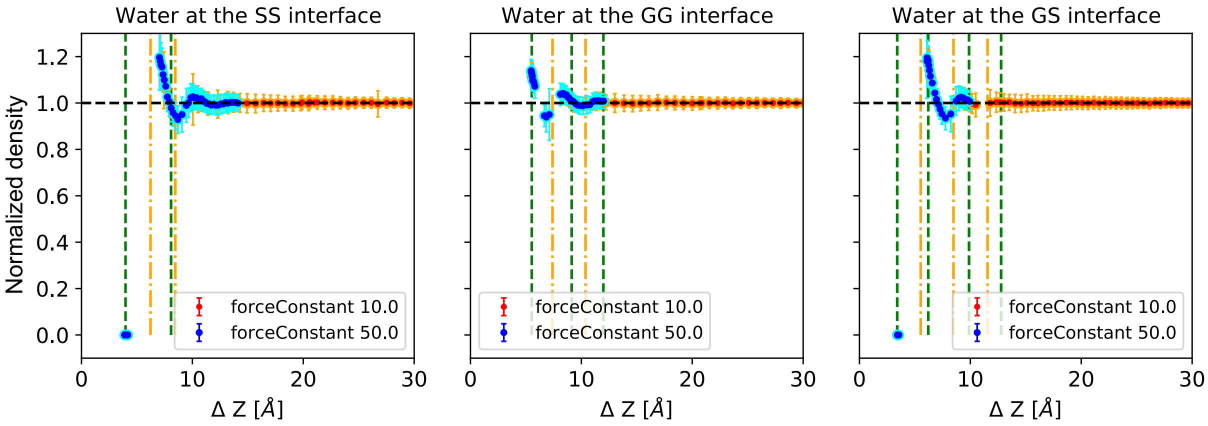

FIG. S11. Average density of water molecules between the two approaching surfaces as a function of the inter-particle distance normalised by the density of water at a far distance. The locations of free energy wells and barriers are displayed in green and orange, respectively. Obtained from simulations with pure water.

S11. HYDROGEN BONDS

In Figure S12 we display the time autocorrelation function (ACF) of hydrogen bonds forming between water and siloxane, gibbsite surfaces. The results show a slow decay of the ACF for the hydrogen bonds forming between water and gibbsite surfaces compared to that of the siloxane (denoted as SS-H_2O-OSi). Noting that GS-H_2O-OAl and GS-H_2O-HOAl denote hydrogen bonds between water molecules and oxygen atoms, -OH groups on the gibbsite surface, respectively. It can be inferred that the formation of hydrogen bond between water molecules and the gibbsite surface is more favourable.

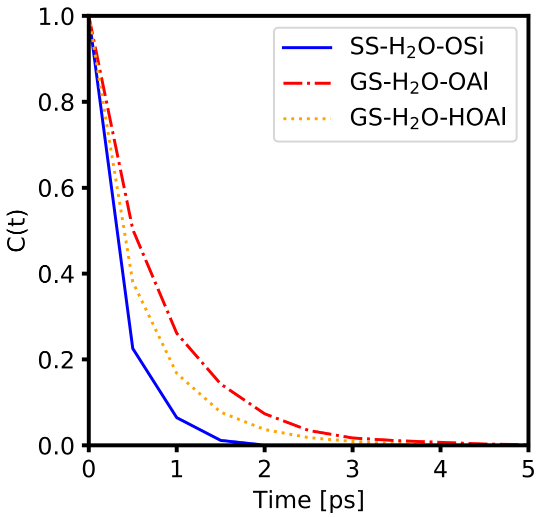

FIG. S12. Time autocorrelation function of hydrogen bonds forming between water molecules and gibbsite, siloxane surfaces.

S12. DIFFUSION COEFFICIENT OF WATER

The self-diffusion of water molecules in two different simulation boxes has been estimated and shown as a function of the coordinate Z in Figure S13. In this calculation, the diffusion coefficient values were calculated using Einstein relation in mean square displacement (MSD) curve for water molecules belonging to a specific slab of $20.0 \times 20.0 \times 3.0$ Å along the Z direction. Points on the left of the figure correspond to water molecules forming the hydration film of one of the nanoparticles. At a distance of more than ca. 1.5 nm the diffusion of water is the same as in bulk. In contrast, close to the kaolinite face the diffusion of water is drastically reduced, by a factor 2.

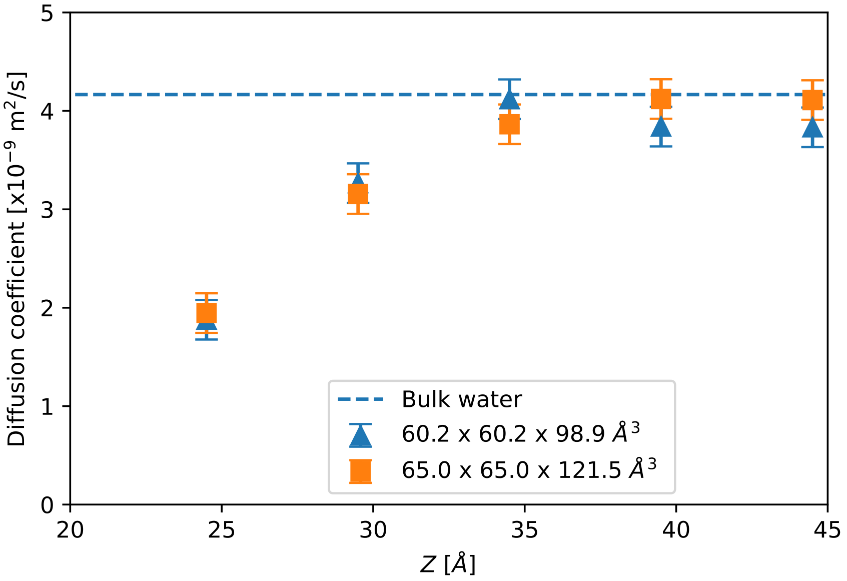

FIG. S13. Diffusion coefficient of water along the Z direction of the simulation box. The results are calculated for two different simulation box sizes, $60.2 \times 60.2 \times 98.9$ Å(orange) and $65.0 \times 65.0 \times 121.5$ Å(blue), and compared to the results of the bulk water at the same condition. The distances between the two particles in these calculations are 30 Å. The results show that diffusion coefficient of water in the middle region between the two particles (around 35-40 Å) reaches the bulk value and the effect of the simulation box size is minimal.

S13. PARTICLE DYNAMICS IN SOLUTION

Particle diffusion in solution is one of the factors determining the kinetics of particle aggregation. Analysis of the umbrella sampling trajectories yields estimates regarding the local diffusion of the kaolinite particles. To extract this information we apply the formalism proposed by Woolf and Roux [23–25]:

$$D(dz = \langle dz \rangle) = \frac{\texttt{Var}(dz)}{\tau_{dz}} \tag{3}$$

where $\langle dz \rangle$ is the average distance along the Z direction between two kaolinite particles under harmonic restraint umbrella sampling simulation at a specific window/distance, $\texttt{Var}(dz) = \langle dz^2 \rangle - \langle dz \rangle^2$ is its variance, and τ_{dz} is its correlation time defined as:

$$\tau_{dz} = \frac{\int_0^\infty \langle \delta dz(t) \delta dz(0) \rangle dt}{\langle \delta dz^2 \rangle}, \tag{4}$$

with $\delta dz(t) = dz(t) - \langle dz \rangle$.

Using this approach, we extract the diffusion coefficients calculated for the kaolinite particle as a function of the distance between the two particles at different orientations in pure and saline water.

---

[1] D. L. Bish, Clays Clay Miner. **41**, 738 (1993).

[2] The crystallographic structure can be obtained from the *Crystallography Open Database.*

[3] M. Pouvreau, J. A. Greathouse, R. T. Cygan, and A. G. Kalinichev, J. Phys. Chem. C **123**, 11628 (2019).

[4] K. Momma and F. Izumi, Journal of Applied Crystallography **44**, 1272 (2011).

[5] T. A. Ho and L. J. Criscenti, Journal of Colloid and Interface Science **600**, 310 (2021).

[6] A. Zen, L. M. Roch, S. J. Cox, X. L. Hu, S. Sorella, D. Alfè, and A. Michaelides, J. Phys. Chem. C **120**, 26402 (2016), 1611.05683.

[7] J. Hutter, M. Iannuzzi, F. Schiffmann, and J. VandeVondele, WIREs Comput Mol Sci **4**, 15 (2013).

[8] J. P. Perdew, K. Burke, and M. Ernzerhof, Phys. Rev. Lett. **77**, 3865 (1996).

[9] J. P. Perdew, K. Burke, and M. Ernzerhof, Phys. Rev. Lett. **78**, 1396 (1997).

[10] S. Grimme, J. Antony, S. Ehrlich, and H. Krieg, The Journal of Chemical Physics **132**, 154104 (2010).

[11] M. Krack, Theor Chem Acc **114**, 145 (2005).

[12] S. Goedecker, M. Teter, and J. Hutter, Phys. Rev. B **54**, 1703 (1996).

[13] J. VandeVondele and J. Hutter, The Journal of Chemical Physics **127**, 114105 (2007).

[14] G. Kresse and J. Hafner, Phys. Rev. B **47**, 558 (1993).

[15] G. Kresse and D. Joubert, Phys. Rev. B **59**, 1758 (1999).

[16] Note that it is fundamental in the ion adsorption to adopt this approach rather than using the energy of the fragments computed separately, due to the uniform compensating background charge that would otherwise be not included in the computation of the free standing slab.

[17] S. Plimpton, J. Comput. Phys. **117**, 1 (1995).

[18] L. Martínez, R. Andrade, E. G. Birgin, and J. M. Martínez, Journal of Computational Chemistry **30**, 2157.

[19] A. I. Jewett, D. Stelter, J. Lambert, S. M. Saladi, O. M. Roscioni, M. Ricci, L. Autin, M. Maritan, S. M. Bashusqeh, T. Keyes, R. T. Dame, J.-E. Shea, G. J. Jensen, and D. S. Goodsell, Journal of Molecular Biology **433**, 166841 (2021), computation Resources for Molecular Biology.

[20] D. T. Limmer, A. P. Willard, P. Madden, and D. Chandler, **110**, 4200 (2013).

[21] A. Phan, D. R. Cole, R. G. Weiß, J. Dzubiella, and A. Striolo, ACS Nano **10**, 7646 (2016).

[22] T. Bui, A. Phan, D. R. Cole, and A. Striolo, The Journal of Physical Chemistry C **121**, 15675 (2017).

[23] T. B. Woolf and B. Roux, Journal of the American Chemical Society **116**, 5916 (1994).

[24] G. Hummer, New Journal of Physics **7**, 34 (2005).

[25] A. Phan, T. Bui, E. Acosta, P. Krishnamurthy, and A. Striolo, Physical Chemistry Chemical Physics **18**, 24859 (2016).
